# Counterfactual curiosity in real decisions: The roles of outcome valence and aging

**DOI:** 10.3758/s13423-024-02569-2

**Published:** 2024-10-25

**Authors:** Alessandro Bogani, Katya Tentori, Benjamin Timberlake, Stefania Pighin

**Affiliations:** Center for Mind/Brain Sciences (CIMeC), University of Trento, Corso Bettini, N. 31, 38068 Rovereto, TN Italy

**Keywords:** Curiosity, Information seeking, Counterfactual thinking, Aging

## Abstract

Non-instrumental counterfactual curiosity (i.e., the search for information about forgone options that is not useful for improving future outcomes) has especially been observed after outcomes perceived as negative and, consequently, attributed to forms of regret management. In three online experiments (*N* = 620), we extended the study of counterfactual curiosity about economically incentivized decisions in younger and older adults. Participants played independent rounds of a card-drawing game by choosing one of two decks to turn over the top, covered card, which could increase, decrease, or have no effect on an initial endowment. Following that, they could examine the top card of the other deck to see if and how the outcome could have differed. Experiment 1 featured identical decks, making the choice between them random. In Experiment 2, participants made a deliberate choice between a riskier and a safer deck, each varying in the extremity of potential wins and losses. In Experiment 3, the decks were identical to those in Experiment 2, but access to counterfactual information was contingent upon participants forfeiting part of their endowment. Results showed a relevant portion of both younger and older adults displayed curiosity for non-instrumental counterfactual information, especially when it was free and likely to reveal that the forgone option would have been better than the chosen one. Older adults exhibited a higher level of curiosity than younger counterparts only when choices were deliberate and counterfactual information was free. These findings are discussed in relation to current perspectives on the regret-management function of counterfactual curiosity.

## Introduction

Individuals have been shown to actively seek non-instrumental information (e.g., Bennett et al., 2016; Bode et al., 2023) – information that cannot be utilized to achieve a more favorable state of the world. For instance, participants presented with sequences of independent probabilistic events (e.g., monetary lotteries akin to the flip of a fair coin) were willing to incur monetary expenses (Bennett et al., 2016), exert physical effort (Goh et al., 2021), or even endure pain (Bode et al., 2023) to receive immediate feedback after each event, even though this information was irrelevant with regard to the likelihood or magnitude of their future outcomes. These and other studies (e.g., Charpentier et al., 2018; Liew et al., 2023; van Lieshout et al., 2021) suggest that the pursuit of information is, thus, not solely driven by its instrumental value, but also by other hedonic (e.g., the emotional response generated by the information) and cognitive (e.g., the reduction of uncertainty related to an outcome) factors, which can, in turn, modulate future behavior (see Matthews et al., 2023). One interesting example of non-instrumental information seeking is the post-decisional tendency to look for forgone (rather than factual) outcomes, a tendency that has been termed *counterfactual curiosity* (FitzGibbon et al., 2021). While this term may have broader application, also encompassing instances in which information-seeking may help improve the likelihood of obtaining better outcomes (Fitzgibbon & Murayama, 2022), in the present work, we limit its usage to cases in which the information is entirely non-instrumental, as these are most closely related to the concept of curiosity (e.g., Gottlieb & Oudeyer, 2018).

Although sparse, research on counterfactual curiosity has consistently shown that people pursue non-instrumental information, especially so when *post-decisional regret* is at stake, that is, in situations in which they suspect they could have experienced a better outcome if they had taken different actions (Shani & Zeelenberg, 2007; Shani et al., 2008; Summerville, 2011; van Dijk & Zeelenberg, 2007). This has led to the hypothesis that counterfactual curiosity may help regulate regret (e.g., Shani & Zeelenberg, 2012; Summerville, 2011). Indeed, the discomfort triggered by experiencing outcomes perceived as negative could be reduced by discovering that the forgone alternative would have been even worse (Shani & Zeelenberg, 2007). But even in cases where counterfactual curiosity reveals that the forgone alternative would have been better than the chosen one, this discovery may still alleviate a negative emotional state. For example, it could show that the forgone alternative was not substantially better than the one experienced (Summerville, 2011) or, at the very least, it could reduce rumination and possible feelings of uncertainty about what could have been (Shani et al., 2008). The few available works on counterfactual curiosity have mainly investigated this topic by asking participants to consider hypothetical scenarios. Importantly, however, counterfactual thoughts have been shown to vary based on whether events are hypothetical or personally experienced (e.g., Girotto et al., 2007; Pighin et al., 2011, 2022). Thus, to gain a more comprehensive understanding, we explored the phenomenon with tasks involving non-instrumental information related to decisions made by the participants themselves. Indeed, to the best of our knowledge, only two published articles have investigated adult counterfactual curiosity in relation to real decisions. In Summerville (2011), participants played multiple independent rounds of a card game, choosing from one of two decks to turn over a covered card that could either increase or decrease their score. After the outcome of their choice was shown, participants had the opportunity to reveal the card they would have drawn from the forgone deck. In line with previous results employing hypothetical scenarios (Shani & Zeelenberg, 2007; Shani et al., 2008), Summerville found that participants were more curious about counterfactual information after negative rather than positive outcomes, that is, in cases in which selecting the forgone deck would have more likely produced a better result. However, the absence of economic incentives (points gained or lost in the game did not affect actual rewards) represents a limitation, situating participants’ decisions somewhere between hypothetical and fully realistic (see also FitzGibbon et al., 2019, for a work on counterfactual curiosity about real but unrewarded decisions in preschool children).

By contrast, an investigation of counterfactual curiosity following real choices can be found in FitzGibbon et al. (2021). In this study, participants engaged in a series of independent trials of a modified version of the Balloon Analogue Risk Task (BART; Lejuez et al., 2002): they had to state the number of pumps with which they wanted to inflate a virtual balloon, knowing that the higher the number of pumps, the greater the monetary reward, but also the greater the risk of bursting the balloon, with the consequent loss of the entire reward. In each trial, the bursting threshold was randomly selected from a uniform distribution between one and 12 pumps. After being informed about whether the number they had chosen caused the balloon to explode or not, participants were given the opportunity to obtain additional information (free or with a cost, depending on the experiment) about the bursting threshold. This information revealed the additional reward participants could have obtained in winning trials or by how much they surpassed the bursting threshold in losing trials, but either way, it was non-instrumental (i.e., useless for maximizing the reward in subsequent trials), as participants were explicitly informed that the bursting threshold was randomly set for each trial. In spite of this, participants expressed interest in the information, after both winning and losing trials, and, to a lesser extent, even when they had to pay for it. This is in line with previous findings, since in these experiments, the counterfactual information conveyed, in the large majority of cases, how a better outcome could have been achieved (i.e., the additional amount that could have been obtained in winning trials and the potential winnings in losing trials).

The present study differs from that of FitzGibbon and colleagues (2021) in at least two respects. First, in their task, counterfactual curiosity was related to a decision characterized by many fine-grained alternatives that could be approximated to a continuous feature, rather than to a decision with two clearly identifiable alternatives (i.e., participants were not required to choose from predefined quantities of pumps, and this allowed them to imagine a number either greater or lesser than the one actually performed). Though limited, previous results suggest that counterfactual thoughts about *continuous* features of a past event are not entirely comparable to those about *categorical* ones, with the former being less readily generated (e.g., Kahneman & Tversky, 1982) and understood (Warren et al., 2023) than the latter. While two previous works on counterfactual curiosity involved making a decision between binary alternatives, they did not feature economically incentivized decisions (FitzGibbon et al., 2019; Summerville, 2011). Therefore, our first objective was to explore counterfactual curiosity in a condition where the information pertains to an economically incentivized decision between two categorical options. Second, and more importantly, in their version of the BART, counterfactual curiosity led mostly to upward-counterfactual scenarios. Specifically, in the large majority of cases, the counterfactual information showed that the obtained outcome was worse than the one they could have obtained, and only occasionally equal to it (when participants stated exactly the number of pumps that maximized the reward). However, in many real-life decisions, it is not obvious how the experienced outcome compares to those of forgone options. Our second goal, thus, was to broaden the investigation of counterfactual curiosity about economically incentivized decisions to situations where the counterfactual information could unveil outcomes of opposite valence (i.e., that could be better or worse than those experienced). Finally, despite research having devoted significant attention to age differences in instrumental information-seeking behaviors (e.g., Levin et al., 2021; Mata & Nunes, 2010; Queen et al., 2012), studies on curiosity-driven information seeking are much less common (for a notable example, see Fastrich et al., 2024). However, gaining a better understanding of how curiosity changes throughout adulthood represents an interesting avenue of research, as maintaining adequate levels of curiosity appears to be essential for successful aging, benefiting cognitive functions such as memory (Galli et al., 2018; McGillivray et al., 2015) and being positively related to survival rates (Swan & Carmelli, 1996; for a review, see Sakaki et al., 2018). Importantly, counterfactual curiosity may change throughout adulthood. Indeed, as people age both the intensity of experienced regret (e.g., Heckhausen et al., 2019; Wrosch & Heckhausen, 2002) and the behavioral response to it (Brassen et al., 2012) appear to decline. If the pursuit of non-instrumental counterfactual information is related to managing regret, younger and older adults might then differ in their tendency to seek such information. Thus, our third aim was to expand the investigation of counterfactual curiosity to include older adults.

## Experiment 1

Experiment 1 was an initial investigation of counterfactual curiosity in which individuals were asked to make economically incentivized decisions by randomly choosing between two identical lottery-like options. Specifically, we explored the impact of outcome valence (negative vs. positive) and of age group (younger vs. older adults) on counterfactual curiosity. Based on previous findings (FitzGibbon et al., 2021; Shani et al., 2008; Summerville, 2011), we expected an increased interest in counterfactual information following negative rather than positive outcomes, as these would more likely induce the suspicion of having missed a better result. Considering the absence of prior age-based studies, any age-related differences were less straightforward to predict. However, if counterfactual curiosity is involved in regret management, we would anticipate an interaction between outcome and age, with differences between younger and older adults being more prominent after negative, potentially regret-inducing outcomes. Specifically, the suggested tendency of older adults to disengage from regret more than younger ones might lead them to exhibit less counterfactual curiosity than their younger counterparts.

### Method

#### Participants

An a priori power analysis, conducted using a simulation approach implemented in R (Green & MacLeod, 2016; Kumle et al., 2021), indicated that a sample of 180 participants would provide 82% power to detect a small-to-medium interaction effect between the outcome experienced and the age group on information seeking. A total of 181 native English-speaking participants from the UK were recruited on Prolific (www.prolific.com). All participants had a Prolific approval rate of at least 90%. Of these, there were 91 younger adults (age = 18–40 years; *M*_age_ = 28.9 years, *SD*_age_ = 6.40 years; 55% females) and 90 older adults (age ≥ 65 years; *M*_age_ = 69.6 years, *SD*_age_ = 3.91 years; 51% females^1^). The upper age limit of 40 years for the sample of younger adults corresponds to the median age of the population of England and Wales in 2021 (Office for National Statistics, 2023), while the lower age limit of 65 years for older adults was based on previous works that investigated the effect of aging on information search (Levin et al., 2021; Queen et al., 2012). In accordance with the hourly payment suggested by Prolific, participants received a base monetary compensation of £0.40, plus any bonus payment won during the experiment.

#### Experimental task and procedure

The task employed in Experiment 1 was an adaptation of the card-drawing game used by Summerville (2011), featuring actual payoffs based on the outcomes of participant choices.

**Fig. 1:**
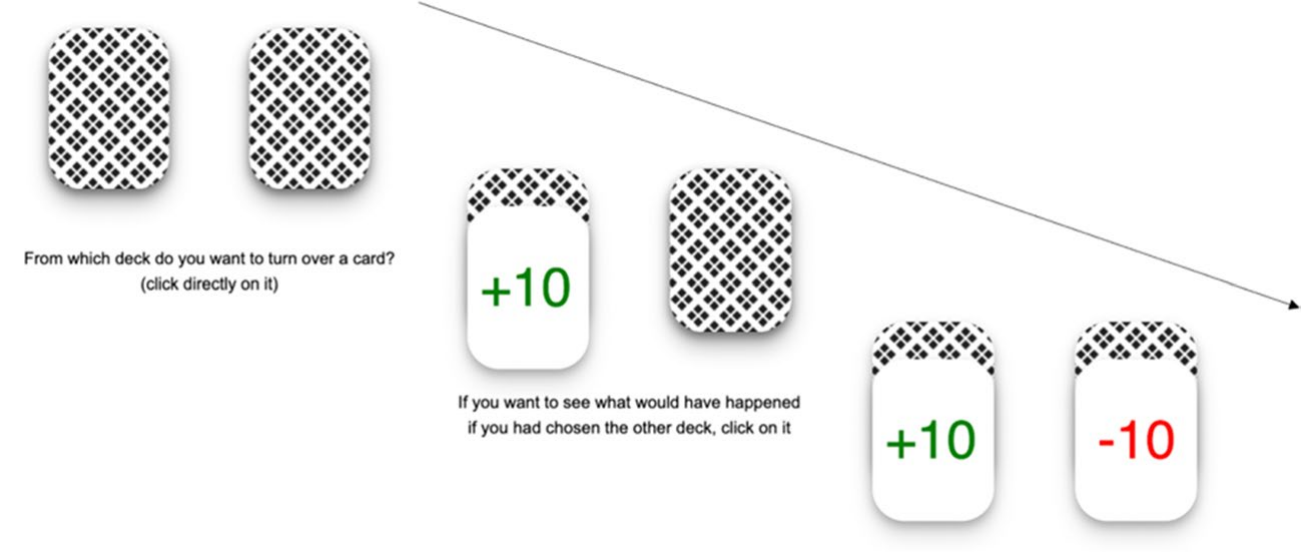
Graphical representation of a round of the card-drawing game used in Experiment 1

At the beginning of the experiment, participants were informed that they would play ten rounds of a card-drawing game using two identical decks (see Fig. 1 for a graphical representation of a round). The instructions explicitly stated that each deck contained a “winning card,” worth +10 pence and a “losing card,” worth -10 pence, which would increase/decrease the 10 pence endowment provided at the beginning of each round. In each round, after the decks had been shuffled, participants selected the deck from which to turn over the top card. The winning and the losing cards had the same probability of appearing at the top of the deck and, thus, of being turned over. After seeing the outcome of their choice, they were offered the opportunity to check the top card of the other deck to see whether they would have won or lost if they had taken the other option. Acquiring counterfactual information about the forgone deck did not involve any monetary cost for participants. Crucially, given the independence of rounds, the counterfactual information about the missed outcome was non-instrumental: participants could not use it to enhance their decision-making in subsequent rounds. Participants were informed that, in addition to their compensation, a bonus payment would be awarded by randomly selecting one of the rounds and honoring the winnings of that round with real money. The total bonus payment they could obtain was either 0 pence (if they turned over a losing card in the round selected for the bonus) or 20 pence (if they turned over a winning card in the selected round). Importantly, before starting the game, the full compositions of both decks (which remained the same throughout the game) were disclosed to participants by showing the cards that made up each deck, side by side, on the screen. They were allowed to inspect the decks for as long as they wished (for the exact wording of the instructions, see the Online Supplementary Material – OSM, available at https://osf.io/6nrtu/). Participants then proceeded to play the game. At the end of the game, when the round selected for the bonus payment was revealed, participants were again offered the opportunity to check what would have happened had they chosen the other deck in that specific round, regardless of whether they had already elected to view the counterfactual information for that round during the game. A playable version of the card-drawing game, along with a template ready to be imported and used on SoSci Survey (an online platform for developing and running online studies; Leiner, 2019), can be found at the following link: https://osf.io/6nrtu/.

#### Statistical analyses

As a preliminary analysis, a logistic regression model was run to assess the comparability of outcomes experienced between the two age groups. Next, a logistic mixed model was fit to evaluate whether counterfactual curiosity (checking vs. not checking the outcome of the forgone deck) was affected by the valence of the outcome experienced (win vs. loss, within-subjects), age group (younger vs. older adults, between-subjects) and their interaction, including random intercepts for participants. In both models, the predictors were effect-coded (reference levels, coded as -1: loss for the outcome experienced; younger adults for the age group). Details of the results of these models (in particular the full list of fixed effects estimates and relative confidence intervals) and of additional analyses can be found in the OSM. These additional analyses comprise the logistic models fit using a Bayesian approach and Bayes factors (presented along with the specific priors used), the logistic models in which covariates were included, and a logistic regression on participants’ counterfactual curiosity in the round that was later randomly selected for the bonus payment. All analyses were performed using R (R Core Team, 2022).

### Results and discussion

**Fig. 2:**
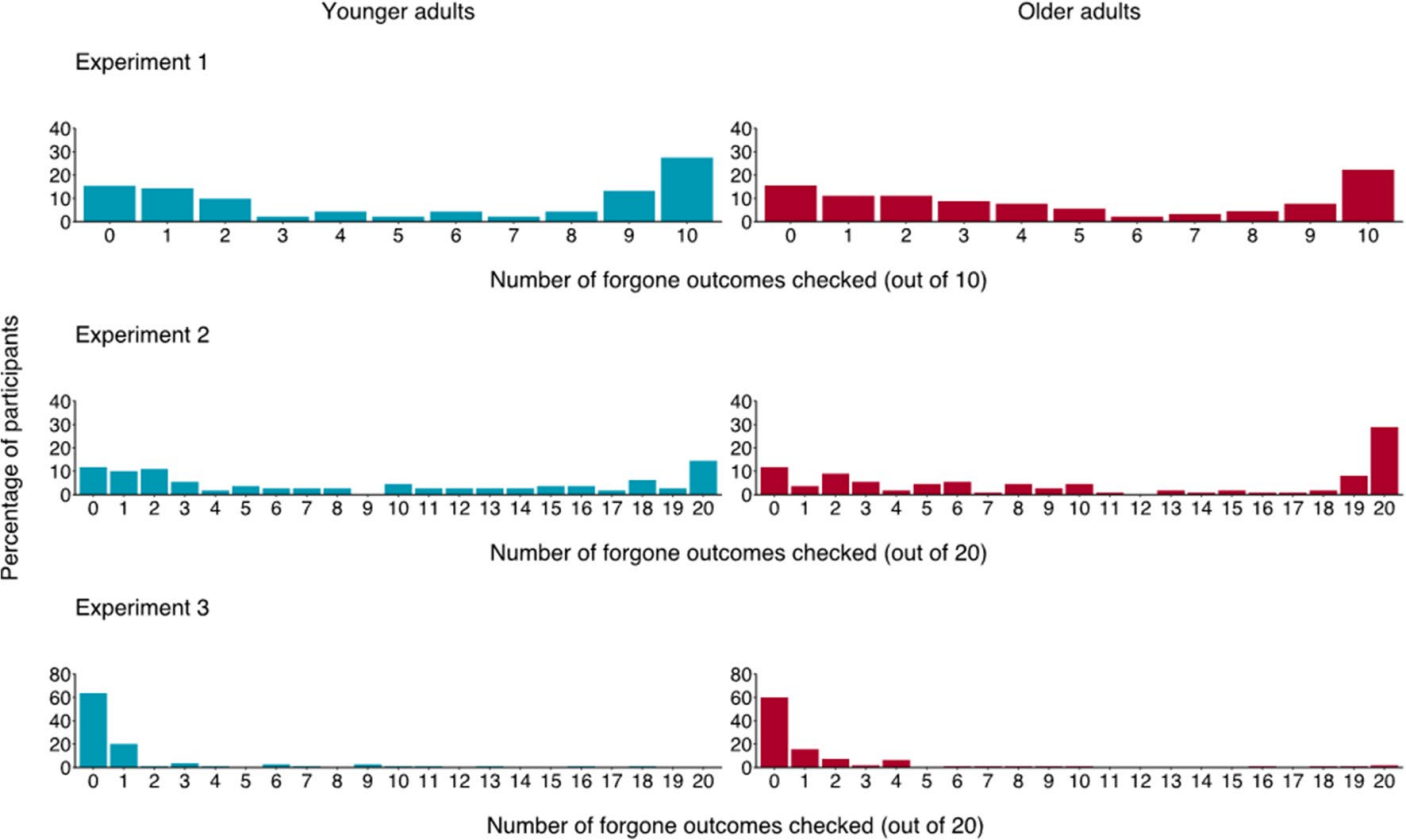
Percentage of rounds in which participants checked the forgone deck in Experiments 1, 2, and 3, by age group

The two age groups experienced comparable outcomes (both obtained 49% losses), *p* = .781, *BF*_*10*_ = 0.05 (see Tables S1–S4 in the OSM for further details about the model). Overall, participants showed substantial interest in the forgone deck, with its outcome checked 51% of the time, and with 85% of participants checking it at least once during the game. Further, the mixed model indicated a significant main effect of the outcome experienced, *OR* = 0.69, *p* < .001 (95% *CI* 0.60–0.79), with the outcome of the forgone deck being checked more frequently after losses (56% of the time) than after wins (47% of the time). No significant effect of age group was observed, *p* = .354, with younger and older adults checking the outcome of the forgone deck 54% and 48% of the time, respectively (see Fig. 2). The interaction between age group and the outcome experienced also failed to reach significance, *p* = .774 (see Tables S5 and S8 in the OSM for details about estimates and relative Bayes Factors).

These results extend previous findings by showing that individuals are also curious about counterfactual information when it informs a categorical rather than a continuous feature of a past event, and when, depending on the outcome experienced, it could portray either better or worse alternative outcomes. Counterfactual information is confirmed to be especially sought after negative outcomes, when individuals have reason to suspect that choosing the forgone deck would have led to a better result. Finally, younger and older adults appeared to hold similar levels of curiosity for non-instrumental information.

## Experiment 2

In Experiment 1, since the two decks were identical, participants did not have any particular reason to choose one over the other, possibly limiting their experience of regret, for which personal responsibility in the decision made is a crucial factor (Zeelenberg & Pieters, 2007). Furthermore, the composition of each deck allowed participants to imagine only two counterfactual possibilities in a given round: after a gain, either a worse outcome or the same one, and after a loss, a better outcome or the same. In order to investigate counterfactual curiosity in a context where regret was potentially more salient and where better or worse alternative scenarios could be envisioned following both positive and negative outcomes, in Experiment 2, we made the choice between the two decks deliberate by differentiating their compositions.

### Method

#### Participants

An a priori power analysis similar to the one conducted for Experiment 1 indicated that a sample of 220 participants would provide 82% power to detect a small-to-medium interaction effect between the valence of the outcome experienced and the age group on information seeking. A new sample of 221 participants was recruited, applying the same inclusion criteria used in Experiment 1. Of these, 110 were recruited among the population of younger adults (*M*_*age*_ = 29.7 years, *SD*_*age*_ = 6.03 years; 59% females^2^) and 111 among the population of older adults (*M*_*age*_ = 69.9 years, *SD*_*age*_ = 4.62 years; 57% females). In accordance with the hourly payment suggested by Prolific, participants received a base monetary compensation of £0.90, plus any bonus payment won during the experiment.

#### Experimental task and procedure

In Experiment 2, a variation of the card-drawing game used in Experiment 1 was employed. In this version, the two decks had the same expected value but differed in their compositions and also included a “neutral card,” which, if drawn, did not affect the endowment. The safer deck consisted of five cards, the outcomes of which (i.e., pence subtracted from or added to the endowment) were -30, -10, 0, +10, +30. The riskier deck also consisted of five cards, but their outcomes were more extreme: -60, -20, 0, +20, +60. To accommodate the worst possible loss, the endowment was 60 pence. This means that, when choosing the riskier deck, participants had the potential to double their endowment (if they turned over a +60 card) but also faced the risk of losing it entirely (if they turned over a -60 card). On the other hand, opting for the safer deck ensured participants a bonus payment of at least 30 pence (if they were to turn over a -30 card). The different compositions of the decks made it possible for participants to contemplate both better and worse counterfactual alternatives after having experienced a negative outcome (e.g., a counterfactual outcome of either +30 or -30 after an actual outcome of -20 obtained as a result of the choice of the riskier deck). Similarly, they could experience not only worse but also better counterfactual alternatives after having experienced a positive outcome (e.g., a counterfactual outcome of either +60 or -60 after an actual outcome of +10 obtained as a result of the choice of the safer deck). The two decks could be presented in either the left or the right part of the screen, with their positions randomly assigned in each round.

**Fig. 3:**
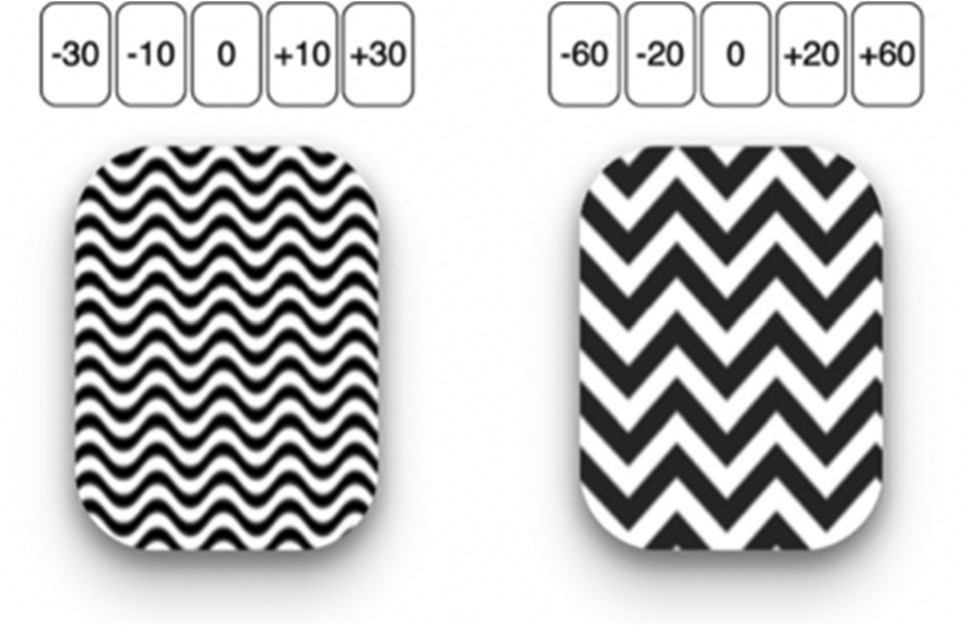
Representation of how the safer (left) and riskier (right) decks appeared to participants in Experiment 2 at the beginning of each round

Instructions were similar to those presented to participants in Experiment 1. Also in this case, we made efforts to ensure clarity regarding the potential payoffs from selecting the riskier or safer decks by allowing participants to inspect their composition for as long as they wished before playing the game. Additionally, to help them remember the cards in each deck, miniatures of each possible card were displayed above the respective decks throughout the game (see Fig. 3). As in Experiment 1, the compositions of the riskier and safer decks were identical in all rounds, irrespective of the outcomes experienced in any preceding round. Thus, in every round, each outcome had the same probability of occurring, making all rounds independent (and, consequently, making the information about the forgone deck non-instrumental). Each participant played 20 rounds. The procedure was identical to that of Experiment 1.

#### Statistical analyses

We conducted statistical analyses similar to those run for Experiment 1, though with a few differences. First, we fit a logistic mixed model to assess whether age group influenced participant tendency to select the riskier or the safer deck. The second difference concerned the experienced outcome, which is one of the predictors of the logistic mixed model on counterfactual curiosity. While in Experiment 1 there were two levels (i.e., win vs. loss), in the model run for Experiment 2, there were ten levels (i.e., ten different outcomes, five for each deck). This variable was treated as categorical rather than continuous to capture possible distinct patterns of information seeking following specific outcomes (e.g., after experiencing a -60 or -30 outcome, that is, the worst possible outcomes in the riskier and safer decks, respectively). Also in this case, the predictors were effect-coded (reference levels, coded as -1: +60 for the outcome experienced; younger adults for the age group). Fixed effects were evaluated by means of a Type-III Wald chi-squared test, and significant effects were explored with Bonferroni-corrected post hoc tests on estimated marginal means.

### Results and discussion

The rate of choosing the riskier deck did not differ between younger and older adults (57% vs. 56%, respectively; *p* = .910, *BF*_*10*_ = 0.09). This made the distributions of outcomes experienced by the two groups comparable (see Tables S12–S15 and Figs. S1 and S2 in the OSM).

**Fig. 4:**
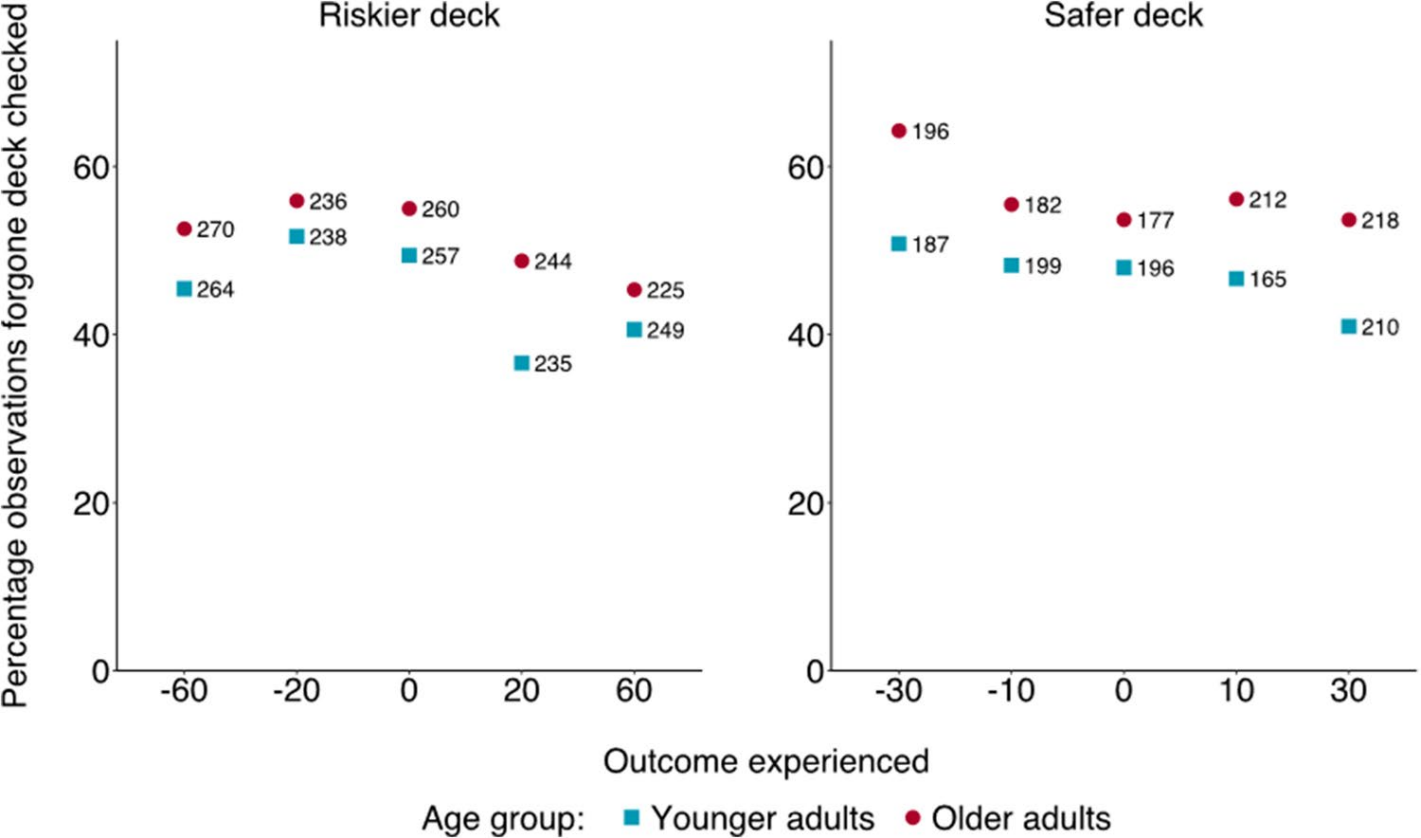
Percentage of observations in Experiment 2 in which the forgone deck was checked, by age group and outcome experienced. Numbers refer to the total observations for a specific combination of age group and outcome experienced (e.g., 270 observations is the tally of older adults turning over a -60 card, and in 53% of those cases the forgone deck was checked)

In line with the findings of Experiment 1, participants checked the outcome of the forgone deck 50% of the time, with 88% of them checking it at least once during the game. The mixed model showed that such tendency was, yet again, much stronger after negative outcomes, *χ*^*2*^(9) = 75.79, *p* < .001. Post hoc tests indicated that, overall, the forgone deck was checked more frequently after experiencing negative or neutral outcomes than after positive ones (see Fig. 4 and Tables S17 and S18 in the OSM). However, as opposed to Experiment 1, the effect of age group was significant, with older adults checking the forgone deck (54%) more than younger ones (46%), *χ*^*2*^(1) = 4.26, *p* = .039. This difference was likely influenced by a subgroup of older adults (29%) who consistently checked the forgone deck in every round (see Fig. 2). The interaction between the valence of the outcome experienced and age group was not significant, *p* = .984 (see Tables S16–S21 in the OSM for details of the model’s estimates and the relative Bayes Factors).^3^

Overall, the results of Experiment 2 confirmed participants’ interest in non-instrumental counterfactual information, even in a task featuring deliberate choices, where both negative and positive outcomes allowed, in most cases, for the contemplation of better and worse alternative scenarios. Similar to Experiment 1, this interest was stronger following a negative outcome, in which a better counterfactual outcome was then more likely to be revealed. Additionally, we found an effect of age group, which consisted of a main effect rather than an interaction, with older adults overall showing greater curiosity about the forgone deck than younger ones did.

## Experiment 3

In Experiments 1 and 2, participants could access counterfactual information about the forgone deck without any monetary cost. In Experiment 3, we gauged the robustness and the generalizability of the results observed in Experiments 1 and 2 by investigating counterfactual curiosity in a condition where participants had to sacrifice part of their monetary endowment to access non-instrumental counterfactual information.

### Method

#### Participants

Since Experiment 3 was identical in terms of materials and structure to Experiment 2, we applied the same inclusion criteria and recruited 220 participants. Two participants in the older adults group were excluded because their self-reported age, contrary to the demographics provided by Prolific, was younger than the cutoff of 65 years.^4^ Thus, the final sample consisted of 218 participants, of whom 110 were recruited from the population of younger adults (*M*_*age*_ = 29.8 years, *SD*_*age*_ = 6.40 years; 54% females^5^) and 108 from the population of older adults (*M*_*age*_ = 69.1 years, *SD*_*age*_ = 3.66 years; 49% females). Participant compensation and bonus payment were the same as those in Experiment 2.

#### Experimental task, procedure, and statistical analyses

The task and procedure in Experiment 3 were identical to those of Experiment 2, with the only exception that participants had to pay 5 pence from their endowment to see what card they would have turned over if they had selected the other deck. To account for possible losses and expenses to acquire the counterfactual information, the endowment provided at the beginning of each round was raised to 65 pence. Thus, in each round, the amount from which participants could decide to subtract 5 pence to inspect the forgone deck depended on the outcome they experienced: when the riskier deck was selected, this amount ranged from a minimum of 5 pence (if the -60 card was turned over) to a maximum of 125 pence (if the +60 card was turned over); when the safer deck was selected, it ranged from a minimum of 35 pence to a maximum of 95 pence (if the -30 or +30 card were turned over, respectively). Importantly, in order to avoid participants misinterpreting the value of the counterfactual information as instrumental, the instructions explicitly stated that, since the rounds of the game are independent, purchasing this information would not have any impact on or yield any insight into subsequent rounds.^6^ Analyses were similar to those performed for Experiment 2.

### Results and discussion

The proportion of trials in which the riskier deck was selected over the safer one was similar for younger (63%) and older (59%) adults (*p* = .303, *BF*_*10*_ = 0.17). Consequently, the two groups experienced a similar distribution of outcomes throughout the game (see Tables S25–S28 and Figs. S3 and S4 in the OSM).

In Experiment 3, the overall percentage of observations in which participants checked the outcome of the forgone deck was substantially lower (7% for younger and 9% for older adults) than in Experiments 1 and 2, with 38% of participants checking it at least once during the game.

**Fig. 5:**
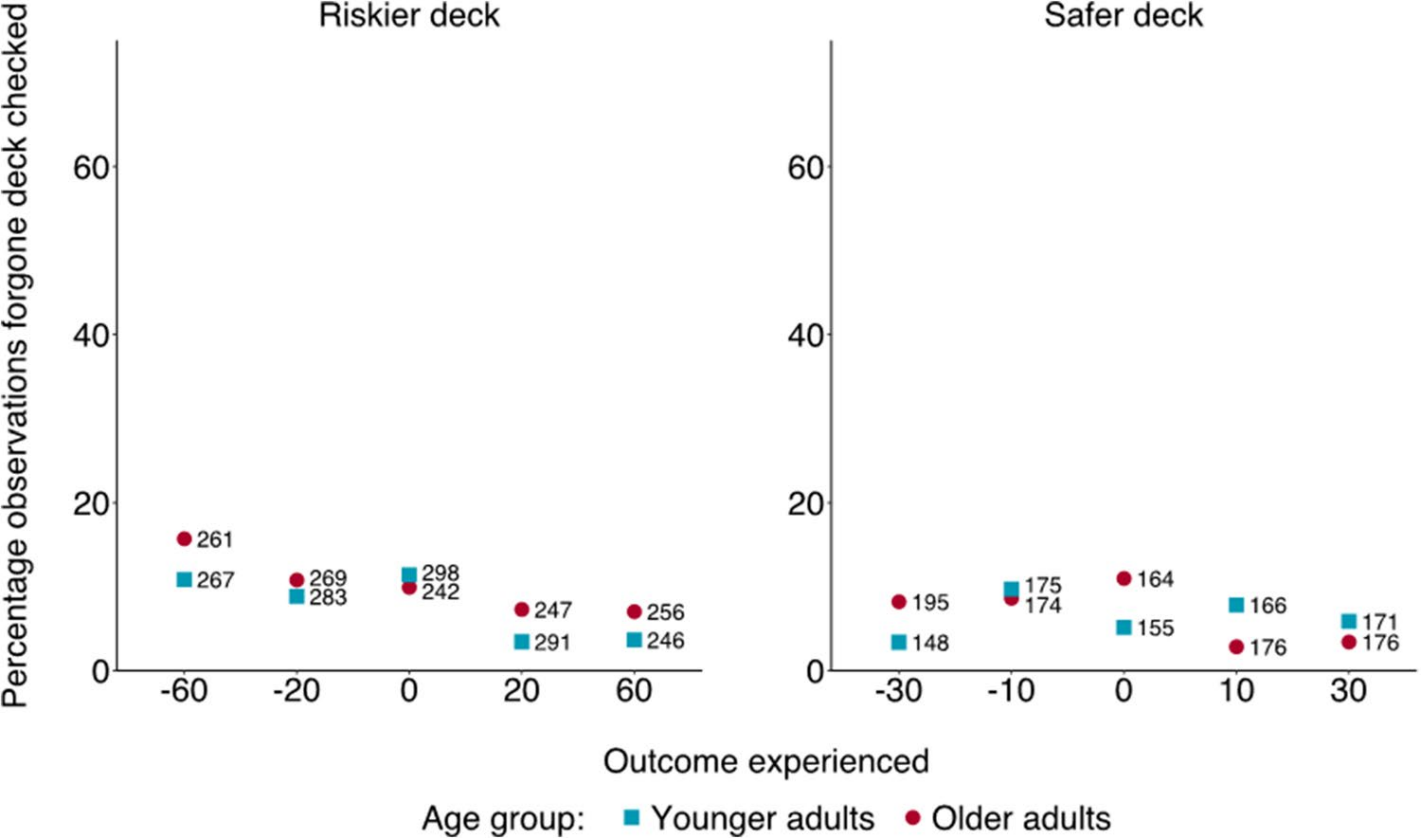
Percentage of observations in which the forgone deck was checked, by age group and outcome experienced in Experiment 3. Numbers refer to the total observations for a specific combination of age group and outcome experienced (e.g., 261 observations is the tally of older adults turning over a -60 card, and in 16% of those cases, the forgone deck was checked)

Neither the effect of age group, *p* = .538, nor its interaction with outcome valence, *p* = .057, were significant. Yet the main effect of outcome persisted, *χ*^*2*^(9) = 61.45, p < .001, with post hoc tests indicating that participants inspected the forgone deck more frequently following negative outcomes, especially after having chosen the riskier deck (see Fig. 5 and Tables S29 to S34 in the OSM for details on the model’s estimates, the relative Bayes Factors, and the post hoc tests).^7^

These results seem to indicate that, for both younger and older adults, interest in non-instrumental counterfactual information is greatly decreased, albeit not eliminated, when access is not free. This is in line with the findings of FitzGibbon et al. (2021) and with participants taking into consideration the fact that the information at issue was non-instrumental.

## General discussion

In the present work, we investigated counterfactual curiosity within the framework of random and deliberate economically incentivized decisions. We used a task that enabled us to expand previous findings with respect to two key dimensions: the information participants could access referred to a categorical, rather than a continuous, feature of a past event, and the information indicated not only the magnitude of the alternative outcome, but also whether it would have been better or worse than the one experienced.

Consistent with previous research (e.g., FitzGibbon et al., 2021; Shani & Zeelenberg, 2007; Shani et al., 2008; Summerville, 2011), our findings revealed a substantial interest in seeking non-instrumental counterfactual information, particularly after negative outcomes, that is, in situations in which it was likely that a different choice would have led to a better result. Nevertheless, this interest was significantly diminished, even if not entirely eliminated, when there was a minimal cost associated with acquiring it.

Overall, our findings are in line with a possible regret-management function of counterfactual curiosity. However, it is worth noting that, in Experiment 2, after turning over a -60 card (i.e., the worst possible outcome) participants still checked the forgone deck 49% of the time. In such situations, counterfactual curiosity cannot serve to positively reappraise the experienced outcome, nor to reduce feelings of discomfort associated with uncertainty (Shani & Zeelenberg, 2012), as individuals already know that all forgone options would have been better than the one experienced. Thus, at least in some cases, individual counterfactual curiosity appears to be better explained by the attempt to reveal a forgone outcome that, although better than the one chosen (e.g., a -30 card after having experienced a -60 one), is still somewhat disappointing, offering, therefore, some degree of relief (for elaboration on this possibility, refer to Summerville, 2011).^8^

The present work also investigated possible age-related differences in counterfactual curiosity. Overall, younger and older adults appeared comparable in terms of counterfactual curiosity, even if the latter were more curious than younger adults when their choice was deliberate and the information was free to access. A possible explanation for this difference is that younger adults, more so than older ones, prioritize maximizing their financial gains in online experiments (see Ryan & Campbell, 2021). In our task, this would see the former checking the forgone deck fewer times so as to finish the experiment as quickly as possible. If that were the case, however, we would have expected a similar age-related effect in Experiment 1, which we did not find. Additionally, both groups exhibited similarly low levels of information seeking when access had a cost, suggesting that the age-related difference was not due to deficits in older adults’ ability to understand the non-instrumental value of the counterfactual information, nor in inhibiting behaviors to satisfy curiosity. The observation that the sole difference between the two age groups manifested in Experiment 2, where counterfactual information was free and regret was presumably more salient than in Experiment 1 (because the choice between the two decks was deliberate rather than random), could at first blush support the idea that aging changes how adults experience and manage this emotion (e.g., Brassen et al., 2012; Tassone et al., 2019; Wrosch & Heckhausen, 2002). However, this explanation does not fully account for our results, as it would predict an interaction between age group and outcome: that is, the two groups should have differed most following negative outcomes, when post-decisional regret is more likely to occur. Instead, in Experiment 2, we found in older adults a generalized increased interest in the forgone deck that was independent of the kind of outcome experienced. If replicated, future studies could evaluate other determinants for this difference between the two age groups. One possibility is that, overall, older adults may find counterfactual scenarios more captivating than younger adults, thus strengthening their motivation to pursue information about what would have happened if they had acted differently. Consistent with this, compared to their younger counterparts, older adults have been reported to rate as more vivid various forms of mental simulations, including counterfactual thinking (De Brigard et al., 2016). Exploring in greater depth how counterfactual curiosity develops throughout adulthood is a compelling avenue for future research, particularly given that maintaining sufficient curiosity has been identified as an important factor in a healthy aging process (Sakaki et al., 2018). Importantly, it must be noted that the present studies involved samples of older adults recruited online through Prolific. Even though various studies have successfully reported age-related differences using similar online samples of older adults (e.g., Byrne et al., 2023; Minton et al., 2024; Sinclair et al., 2021), it remains unclear to what extent online samples of older participants differ from those typically tested in the laboratory – or even more, from the general population – particularly in terms of proficiency with technology (Greene & Naveh-Benjamin, 2022; Turner et al., 2020).

In conclusion, our study builds upon previous findings in counterfactual curiosity by investigating a diverse set of experimental conditions applied to two distinct populations of participants. These conditions involve the search for non-instrumental information related to real decisions under varying degrees of uncertainty. Future studies could enhance our understanding of this phenomenon by exploring in greater depth the effects of the characteristics of the tasks. For example, a promising avenue of research would be to better explore how counterfactual curiosity changes when varying the degree of participant agency in determining the experienced outcome: from outcomes completely determined by external factors (in which only disappointment can be experienced) to outcomes resulting from deliberate personal choices (in which regret becomes available) and finally to performance-related outcomes (i.e., in which individual engagement and competence may contribute to determining the experienced outcome and in which regret may be significantly enhanced). It would also be interesting to explore in greater detail how gradual increments and decrements in the cost to acquire information about forgone outcomes might change the propensity to exercise counterfactual curiosity. This would help to understand whether non-instrumental counterfactual curiosity is observed only when people perceive the possible costs associated with it as negligible. Overall, these possible investigations would enhance our understanding of this phenomenon by identifying specific contexts where it is more likely to be observed and the factors influencing such information-seeking behavior.

## Data Availability

The data availability statement was incorporated in the open practices statement.

## Footnotes

1 For one participant in the older-adult group, information about gender was not available.

2 One participant in the younger-adult group opted not to disclose information about their gender.

3 Results did not change when the experienced outcome was included in the model as a continuous rather than a categorical variable.

4 This type of exclusion was not performed in Experiments 1 and 2 because in those a self-report question on age was not included.

5 One participant in the younger-adult group opted not to disclose information about their gender.

6 As opposed to Experiment 2, we removed the opportunity to inspect the forgone deck in the replay of the round selected to compute the bonus payment. Indeed, in Experiment 2, analyses of information seeking in that replica round yielded unreliable results (see OSM). This was likely due to the low number of observations in several cells resulting from the intersection of the age-group and outcome-experienced factors (e.g., older adults experiencing a +60).

7 Results did not change when the experienced outcome was included in the model as a continuous rather than a categorical variable.

8 This explanation assumes that participants correctly considered the information about the forgone outcome as non-instrumental. An anonymous reviewer noted that, although there is objectively no doubt that the information in question was non-instrumental and that appropriate steps were taken to design the experiments to ensure participants understood it as such, a minority of participants who consistently checked the forgone deck in all rounds of the game (25%, 22%, and 1% in Experiments 1, 2, and 3, respectively) may nonetheless have mistakenly assigned some value to it. Future research might consider controlling for this possibility with dedicated experimental setups.

## Declarations

### Open practices statement

Materials, anonymized data, analysis scripts and Supplementary Materials for all experiments are available via the Open Science Framework at https://osf.io/6nrtu/. None of the experiments were preregistered.
